# Hesperetin-7,3'-*O*-dimethylether selectively inhibits phosphodiesterase 4 and effectively suppresses ovalbumin-induced airway hyperresponsiveness with a high therapeutic ratio

**DOI:** 10.1186/1423-0127-18-84

**Published:** 2011-11-11

**Authors:** You-Lan Yang, Hsin-Te Hsu, Kuo-Hsien Wang, Cheng-Ying Han, Chien-Ming Chen, Chi-Ming Chen, Wun-Chang Ko

**Affiliations:** 1 School of Respiratory Therapy, College of Medicine, Taipei Medical University, 250 Wu-Hsing St., Taipei 110, Taiwan; 2 Department of Otolaryngology, Taipei Medical University Hospital, 252 Wu-Hsing St., Taipei 110, Taiwan; 3 Department of Dermatology, Taipei Medical University Hospital, 252 Wu-Hsing St., Taipei 110, Taiwan; 4 Department of Pharmacology, College of Medicine, Taipei Medical University, 250 Wu-Hsing St., Taipei 110, Taiwan; 5 Department of Medical Technology, College of Medicine, Taipei Medical University, 250 Wu-Hsing St., Taipei 110, Taiwan; 6 Department of Medicinal Chemistry, College of Pharmacy, Taipei Medical University, 250 Wu-Hsing St., Taipei 110, Taiwan

**Keywords:** Airway hyperresponsiveness, allergic asthma, chronic obstructive pulmonary disease, cytokine, hesperetin-7,3'-*O*-dimethylether, phosphodiesterase-4 inhibitor

## Abstract

**Background:**

Hesperetin was reported to selectively inhibit phosphodiesterase 4 (PDE4). While hesperetin-7,3'-*O*-dimethylether (HDME) is a synthetic liposoluble hesperetin. Therefore, we were interested in investigating its selectivity on PDE4 and binding ability on high-affinity rolipram-binding sites (HARBs) *in vitro*, and its effects on ovalbumin-induced airway hyperresponsiveness *in vivo*, and clarifying its potential for treating asthma and chronic obstructive pulmonary disease (COPD).

**Methods:**

PDE1~5 activities were measured using a two-step procedure. The binding of HDME on high-affinity rolipram-binding sites was determined by replacing 2 nM [^3^*H*]-rolipram. AHR was assessed using the FlexiVent system and barometric plethysmography. Inflammatory cells were counted using a hemocytometer. Cytokines were determined using mouse T helper (Th)1/Th2 cytokine CBA kits, and total immunoglobulin (Ig)E or IgG_2a _levels were done using ELISA method. Xylazine (10 mg/kg)/ketamine (70 mg/kg)-induced anesthesia was performed.

**Results:**

HDME revealed selective phosphodiesterase 4 (PDE4) inhibition with a therapeutic (PDE4_H_/PDE4_L_) ratio of 35.5 *in vitro*. *In vivo*, HDME (3~30 μmol/kg, orally (p.o.)) dose-dependently and significantly attenuated the airway resistance (R_L_) and increased lung dynamic compliance (C_dyn_), and decreased enhanced pause (P_enh_) values induced by methacholine in sensitized and challenged mice. It also significantly suppressed the increases in the numbers of total inflammatory cells, macrophages, lymphocytes, neutrophils, and eosinophils, and levels of cytokines, including interleukin (IL)-2, IL-4, IL-5, interferon-γ, and tumor necrosis factor-α in bronchoalveolar lavage fluid (BALF) of these mice. In addition, HDME (3~30 μmol/kg, p.o.) dose-dependently and significantly suppressed total and ovalbumin-specific immunoglobulin (Ig)E levels in the BALF and serum, and enhanced IgG_2a _level in the serum of these mice.

**Conclusions:**

HDME exerted anti-inflammatory effects, including suppression of AHR, and reduced expressions of inflammatory cells and cytokines in this murine model, which appears to be suitable for studying the effects of drugs on atypical asthma and COPD, and for screening those on typical asthma. However, HDME did not influnce xylazine/ketamine-induced anesthesia. Thus HDME may have the potential for use in treating typical and atypical asthma, and COPD.

## Background

Phosphodiesterases (PDEs) are classified according to their primary protein and complementary (c)DNA sequences, co-factors, substrate specificities, and pharmacological roles. It is now known that PDEs comprise at least 11 distinct enzyme families that hydrolyze adenosine 3',5' cyclic monophosphate (cAMP) and/or guanosine 3',5' cyclic monophosphate (cGMP) [1]. PDE1~5 isozymes, which are calcium/calmodulin dependent (PDE1), cGMP stimulated (PDE2), cGMP inhibited (PDE3), cAMP specific (PDE4), and cGMP specific (PDE5), were found to be present in the canine trachea [2], guinea pig lungs [3], and human bronchi [4]. PDE3 and PDE4 were identified in the guinea pig airway [5], but other isozymes might also be present. PDE4 may adopt two different conformations which have high (PDE4_H_) and low (PDE4_L_) affinities for rolipram, respectively. In general, it is believed that inhibition of PDE4_H _is associated with adverse responses, such as nausea, vomiting, and gastric hypersecretion, while inhibition of PDE4_L _is associated with anti-inflammatory and bronchodilating effects. Therefore the therapeutic ratio of selective PDE4 inhibitors for use in treating asthma and chronic obstructive pulmonary disease (COPD) is defined as the PDE4_H_/PDE4_L _ratio [6,7].

Hesperetin (5,7,3'-trihydroxy-4'-methoxyflavanone), one of the most-common flavonoids in *Citrus*, is also present in herbal medicine as glycosides. For example, hesperidin and neohesperidin are abundantly present in the fruit peel of *Citrus aurantium *L. (Rutaceae), a well-known traditional Chinese medicine (TCM) called "Chen-Pi"; they are used as an expectorant and stomach tonic, and contain vitamin P, a remedy for preventing capillary fragility and hypertension [8]. These glycosides are easily hydrolyzed by glycosidase to form hesperetin after ingestion. Based on lung histopathological studies using hematoxylin and eosin and alcian blue-periodic acid-Schiff staining, hesperidin was recently reported to inhibit inflammatory cell infiltration and mucus hypersecretion compared with the ovalbumin-induced group of mice in a murine model of asthma [9]. Men with higher hesperetin intake have lower mortality from cerebrovascular disease and lung cancer, and lower incidences of asthma [10]. Because hesperetin was reported to selectively inhibit PDE4 activity [11], it was used as a lead compound to synthesize hesperetin-7,3'-*O*-dimethylether (HDME), a more-liposoluble derivative of hesperetin. Therefore, we were interested in investigating the PDE4_H_/PDE4_L _ratio and suppressive effects of HDME on ovalbumin (OVA)-induced airway hyperresponsiveness (AHR), and clarifying its potential for treating asthma and COPD. Although both asthma and COPD are associated with an underlying chronic inflammation of the airways, there are important differences with regard to the inflammatory cells and mediators involved. The key inflammatory cells in COPD are macrophages, CD8+ T-lymphocytes and neutrophils. Macrophages are strongly increased in the airway lumen, lung parenchyma and bronchoalveolar lavage fluid. In the airway wall and lung parenchyma, the ratio of CD8+/CD4+ T-lymphocytes increases. Neutrophils are increased in sputum and their number grows with the progression of the disease. In contrast, the key inflammatory cells in asthma are mast cells, eosinophils and CD4+ T-lymphocytes. Both diseases are sensitive to steroids. However, COPD shows a limited response to inhaled corticosteroids as compared to the efficacy achieved in asthma. Owing to the side effects of steroids, other therapeutics such as selective PDE4 or dual PDE3/4 inhibitors are developing. However, these developing inhibitors are also limited for the use of asthma and COPD in clinic because of their emetic side effect. This side effect can be easily assessed in non-vomiting species, such as rats or mice, in which selective PDE4 inhibitors reduce the duration of xylazine/ketamine-induced anesthesia [12,13].

## Materials and methods

### Reagents and animals

HDME (mol wt., 330.27) was synthesized according to a previous method [14] in our laboratory and identified by spectral methods, including ultraviolet, infrared, mass spectroscopy, and nuclear magnetic resonance spectroscopic techniques. The purity of the compound exceeded 98% as determined by high-performance liquid chromatography. OVA, methacholine (MCh), aluminum sulfate hexadecahydrate, dimethylsulfoxide (DMSO), chloralose, urethane, Tris-HCl, Bis-Tris, benzamidine, phenylmethanesulfonyl fluoride (PMSF), *d,l*-dithiothreitol, polyethyleneimine, ethylenediaminetetraacetic acid (EDTA), bovine serum albumin (BSA), cAMP, cGMP, calmodulin, Dowex resin, *Crotalus atrox *snake venom, xylazine, and ketamine were purchased from Sigma Chemical (St. Louis, MO, USA). Vinpocetine, *erythro*-9-(2-hydroxy-3-nonyl)-adenine HCl (EHNA), milrinone, 4-(3-butoxy-4-methoxybenzyl)-2-imidazolidinone (Ro 20-1724), and Zaprinast were purchased from Biomol (Plymouth Meeting, PA, USA). Freund's adjuvant (*Mycobacterium butyricum*) was purchased from Pierce Biotechnology (Rockford, IL, USA). Mouse Th1/Th2 cytokine CBA kits, and mouse IgE enzyme-linked immunosorbent assay (ELISA) sets were purchased from Pharmingen (San Diego, CA, USA). Ethyl alcohol and polyethylene glycol (PEG) 400 were purchased from Merck (Darmstadt, Germany). [^3^*H*]-cAMP, [^3^*H*]-cGMP, and [*methyl*-^3^*H*]-rolipram were purchased from Amersham Pharmacia Biotech (Buckinghamshire, UK). Other reagents, such as CaCl_2_, MgCl_2_, and NaCl, were of analytical grade. HDME and Ro 20-1724 were dissolved in a mixture of ethyl alcohol and DMSO (1: 1). The vehicle, a mixture of DMSO: ethyl alcohol: PEG 400: saline (0.5: 0.5: 1: 8, v/v) used *in vivo *studies had no abnormal behavior in mice after oral administration. Other reagents were dissolved in distilled water.

Male Hartley guinea pigs (500~600 g) and female BABL/c mice at 8~12 weeks old were purchased from the Animal Center of the National Science Council (Taipei, Taiwan), and housed in ordinary cages at 22 ± 1°C with a humidity of 50%~60% under a constant 12/12-h light/dark cycle and provided with food and water *ad libitum*. Under a protocol approved by the Animal Care and Use Committee of Taipei Medical University, the following *in vivo *and *in vitro *experiments were performed.

### Competitive inhibition of PDE1, PDE3, and PDE4 activities

Activities of PDE1~5 in the homogenate of guinea pig lungs or hearts were measured by a two-step procedure according to the previous method [15], using cAMP with [^3^*H*]-cAMP or cGMP with [^3^*H*]-cGMP as substrates. In the Lineweaver-Burk analysis, the reaction mixture contained 10 μl of vehicle or inhibitors, at various concentrations of HDME or selective PDE1, PDE3, and PDE4 inhibitors, such as vinpocetine [16], milrinone [17], and Ro 20-1724 [18] as reference drugs. The reagents and homogenate were mixed on ice, and the reaction was initiated by transferring the mixture to a water bath at 37°C. Following a 30-min incubation, the reaction was stopped by transferring the reaction vessel to a bath of boiling water for 3 min. After cooling on ice, 20 μl of a 1 mg/ml solution of *Crotalus atrox *snake venom was added to the reaction mixture, and the mixture was incubated at 37°C for 10 min. Unreacted [^3^*H*]-cAMP or [^3^*H*]-cGMP was removed by the addition of 500 μl of a 1-in-1 Tris-HCl (40 mM) buffer suspension of Dowex resin (1 × 8-200) with incubation on ice for 30 min. Each tube was then centrifuged at 3700 *g *for 2 min, and 150 μl of the supernatant was removed for liquid scintillation counting. Less than 10% of the tritiated cyclic nucleotide was hydrolyzed in this assay. The total protein in each fraction used was assayed according to a previous method [19]. PDE activities are reported as nmol/mg/min.

### Determination of PDE4_H _values

When the above-mentioned guinea pigs were sacrificed, the whole brains were removed and homogenized with a glass/Teflon homogenizer (Glas-Col, Terre Haute, IN, USA) in 10 volumes of cold medium (pH 6.5) containing 20 mM Bis-Tris, 2 mM benzamidine, 2 mM EDTA, 50 mM sodium chloride, 0.1 mM PMSF, and 1 mM dithiothreitol. At 4°C, the homogenate was centrifuged at 170 *g *for 5 min to remove connective tissues and blood vessels. The suspended homogenate was then re-centrifuged at 40,000 *g *for 30 min to separate the cytosolic and particulate portions. The particulate portion was re-suspended in a suspension at a concentration of 400 mg/ml (wet weight/volume), after washing three times with homogenizing buffer. The particulate portion mainly consisted of cell membranes. The binding ability of HDME (3~300 μM) to high-affinity rolipram-binding sites (HARBSs) of guinea pig brain cell membranes was determined by replacing 2 nM [^3^*H*]-rolipram in a reaction buffer at 30°C for 1 h, according to the method described by previous investigators [20,21] and modified by us. Briefly, the reaction buffer consisted of 50 mM Tris-HCl and 5 mM MgCl_2 _(pH 7.5). The total volume of the reaction mixture was 25 μl, consisting of 10 μl of the particulate suspension, 10 μl of [^3^*H*]-rolipram, and 5 μl of HDME or Ro 20-1724 (1~10,000 nM), a reference drug. After 1 h, the reaction was terminated by moving the reaction vessel into crushed ice. Then the reaction mixture was transferred onto Whatman GF/B glass-fiber filters, which were soaked in a 0.3% polyethyleneimine solution in a mini-funnel. The reaction mixture was filtered by centrifugation at 90 *g *for 10 s, and the filtrate was collected into a 1.5-ml Eppendorf tube with the top adapted to the outlet of the mini-funnel. The filters were washed with 300 μl of reaction buffer three times each in the same way, and transferred into 2 ml of cocktail for radiation counting (total binding) using a β-scintillation counter (Beckman, Fullerton, CA, USA). Non-specific binding, which was defined in the presence of 10 μM Ro 20-1724, was subtracted from total binding to yield specific binding. Effective concentration (EC_50_) values of HDME and Ro 20-1724, at which a half of the [^3^*H*]-rolipram that was bound onto HARBSs of cell membranes was displaced, were defined as PDE4_H _values, and these were related to any adverse effects, such as nausea, vomiting, and gastric hypersecretion [7].

### Airway hyperresponsiveness (AHR) in vivo

**Figure 1:**
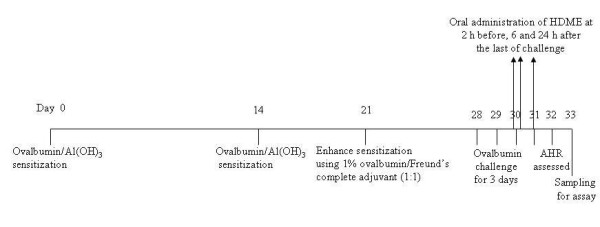
**The schedule of sensitization, challenge and drug administration in mice**. AHR, airway hyperresponsiveness; Al(OH)_3_, aluminum hydroxide gel; HDME, hesperetin-7,3'-*O*-dimethylether.

According to the schedule (Figure 1), ten female BABL/c mice in each group were sensitized by an intraperitoneal (i.p.) injection of 20 μg of OVA emulsified in 2.25 mg of an aluminum hydroxide gel, prepared from aluminum sulfate hexadecahydrate, in a total volume of 100 μl on days 0 and 14. On day 21, these mice were injected with (i.p.) 100 μl of a mixture of 1% OVA and Freund's complete adjuvant (1:1). Mice were challenged *via *the airway using 1% OVA in saline for 30 min on days 28, 29, and 30 by ultrasonic nebulization. After the last of OVA challenges [22], AHR was assessed on day 32 (48 h after 1% OVA provocation) in each group. Each group of mice was orally (p.o.) administered the vehicle (control) or 3~30 μmol/kg of HDME 2 h before and 6 and 24 h after OVA provocation. For comparison, sham-treated mice were challenged with saline instead of 1% OVA (non-challenged). The vehicle, a mixture of DMSO: ethyl alcohol: PEG 400: saline (0.5: 0.5: 1: 8, v/v), or HDME was administered (p.o.) at a volume of 0.01 ml/g of body weight. AHR was assessed using two methods: (1) in anesthetized ventilated mice, AHR was assessed as previously described [23] by measuring changes in the airway resistance (R_L, _cmH_2_O/ml/sec) and lung dynamic compliance (C_dyn_, ml/cmH_2_O) after challenge with aerosolized methacholine (MCh, 0.78~25 mg/ml) using the FlexiVent system (SCIREQ, Montreal, Quebec, Canada). Anesthetized (urethane 600 mg/kg and chloralose 120 mg/kg, i.p.), tracheostomized (stainless-steel cannula, 18 G) mice were mechanically ventilated (at 150 breaths/min, with a tidal volume of 10 ml/kg, positive end-expiratory pressure of 3 cmH_2_O). (2) in unrestrained animals by barometric plethysmography [24] using a whole-body plethysmograph (WBP) and analyzed using software of Life Science Suite P3 Analysis Modules (Gould, LDS Test and Measurement LLC, Valley View, OH, USA). Mice were placed into the main chamber of the WBP, and the baseline enhanced pause (P_enh_) value was determined. Then mice were first nebulized with phosphate-buffered saline (PBS), and subsequently with increasing doses (6.25~50 mg/ml) of MCh for 3 min for each nebulization, followed by readings of breathing parameters for 3 min after each nebulization to determine P_enh _values.

### Inflammatory cells, cytokines and immunoglobulins

Twenty-four hours after P_enh _determination, these mice were anesthetized with pentobarbital (50 mg/kg, i.p.), and the lungs were lavaged *via *a tracheal tube with PBS (1 × 1.0 ml, 37°C). After lavage, blood was collected from the jugular vein and allowed to sit so that it would coagulate. The collected bronchoalveolar lavage fluid (BALF) and coagulated blood were respectively centrifuged at 630 *g *for 7 min and at 3700 *g *for 10 min at 4°C. After centrifugation, the BALF and serum supernatants were stored at -20°C until determination of cytokines, including interleukin (IL)-2, IL-4, IL-5, tumor necrosis factor (TNF)-α, and interferon (IFN)-γ by flow cytometric methods [25] using mouse T helper (Th)1/Th2 cytokine CBA kits, and of total immunoglobulin (Ig)E or IgG_2a _using ELISA kits (Pharmingen, San Diego, CA, USA) according to the respective recommendations of the manufacturers. OVA-specific IgE was measured as described previously [26]. Wells were coated with 100 μl of OVA (20 μg/ml) instead of the capture antibody. Levels are expressed in arbitrary units, where 1 arbitrary unit equals the optical density of the sample divided by the optical density of unchallenged mouse serum or BALF (standard). The BALF pellet was resuspended in ACK lysing buffer (1.658 g NH_4_Cl, 0.2 g KHCO_3 _and 1.44 mg EDTA in 200 ml of water) to lyse the residual erythrocytes in each sample. The number of inflammatory cells was counted using a hemocytometer (Hausser Scientific, Horsham, PA, USA). Cytospun slides were stained and differentiated in a blinded fashion by counting at least 100 cells under light microscopy. All undetectable data (< 1 pg/ml) of cytokines were taken as 0 pg/ml.

### Xylazine/ketamine-induced anesthesia

According to a previously described method [13] and modified by us, HDME (10~100 μmol/kg, subcutaneously (s.c.)) or Ro 20-1724 (0.01~1 μmol/kg, s.c.), a reference drug, was respectively injected into 8~12-week-old female BALB/c mice 1 or 0.25 h prior to an i.p. injection of xylazine (10 mg/kg)/ketamine (70 mg/kg). The vehicle (control) for HDME or Ro 20-1724 was a mixture of DMSO: ethyl alcohol: PEG 400: saline (0.5: 0.5: 1: 8, v/v). After loss of the righting reflex (i.e., when a mouse remained on its back and no longer spontaneously righted itself to a prone position), the duration of anesthesia was measured until its return as the endpoint [13].

### Statistical methods

All values are given as the means ± SEM. Differences among values were statistically calculated by one-way analysis of variance (ANOVA), and then determined by Dunnett's test. The difference between two values, however, was determined by the use of Student's *t*-test. Differences with *p *< 0.05 were considered statistically significant.

## Results

### Competitive inhibition of PDE1, PDE3, and PDE4 activities

**Figure 2:**
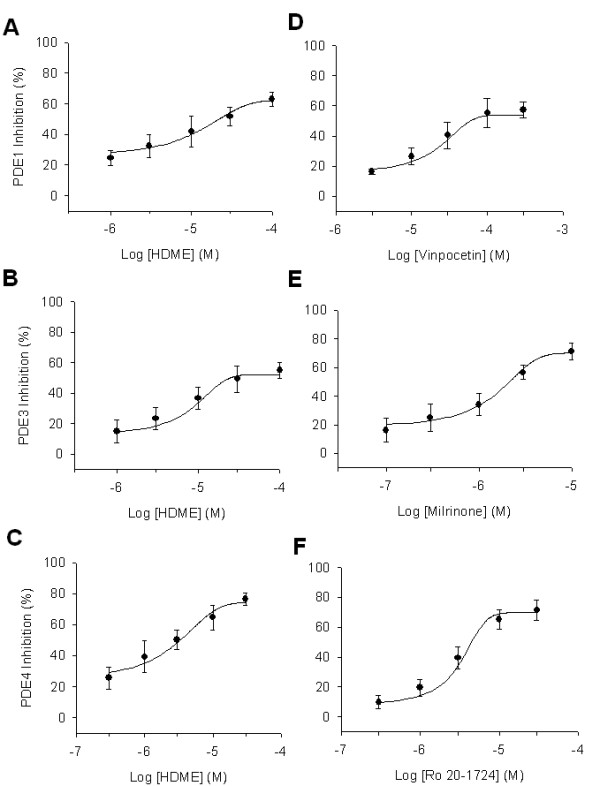
**Log concentration-inhibition curves**. Log concentration-inhibition curves of HDME (A-C) and reference drugs (D-F) on PDE1 (A, D), PDE3 (B, E), and PDE4 (C, F) activities.

**Figure 3:**
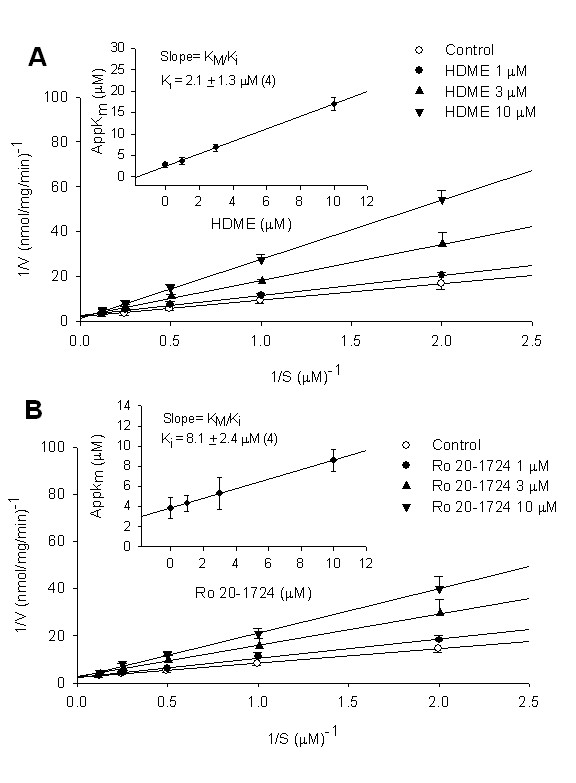
**Inhibition of PDE4-induced cAMP hydrolysis by HDME (A) and Ro 20-1724 (B)**. Activities of PDE4 in the presence of various concentrations of HDME or Ro 20-1724, and the substrate (cAMP) were plotted according to a Lineweaver-Burk analysis. K_i _was determined from the equation of the apparent K_m _as a function of the inhibitor concentration (inset). Each value represents the mean ± SEM (*n *= 3).

HDME did not inhibit PDE2 or PDE5 activities (IC_50 _value > 100 μM), but it concentration-dependently inhibited PDE1, PDE3, and PDE4 activities with respective IC_50 _values of 22.1 ± 6.4 (*n *= 4), 24.6 ± 3.5 (*n *= 4), and 3.0 ± 0.9 μM (*n *= 4) (Figure 2A-C). Similarly, the reference drugs, vinpocetin, milrinone, and Ro 20-1724, inhibited these enzymes with respective IC_50 _values of 42.3 ± 5.8 (*n *= 6), 2.5 ± 1.6 (*n *= 5), and 4.3 ± 2.1 μM (*n *= 4) (Figure 2D-F). The IC_50 _value of HDME for PDE4 inhibition was significantly less than those for PDE1 and PDE3 inhibition. According to the Lineweaver-Burk analysis, HDME (1~10 μM) and Ro 20-1724 (1~10 μM) competitively inhibited PDE4 activity (Figure 3), with calculated dissociation constant for inhibitory binding (K_i_) values of 2.1 ± 1.3 (n = 4) and 8.1 ± 2.4 (*n *= 4) μM, respectively (Figure 3 inset).

### PDE4_H_/PDE4_L _ratios

**Figure 4:**
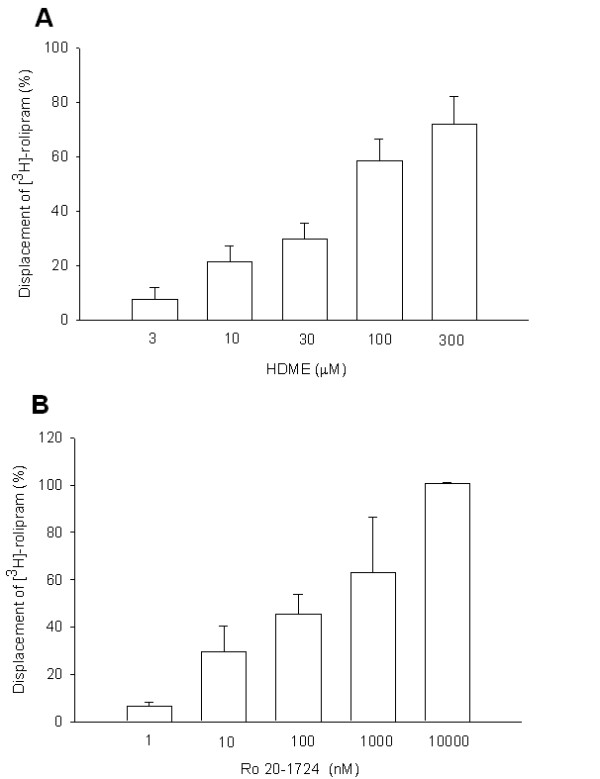
**Displacement of [^3^H]-rolipram by HDME**. Displacement of [^3^H]-rolipram by HDME (A) and Ro 20-1724 (B) in high-affinity rolipram binding sites of guinea pig brain particulate. Each value represents the mean ± SEM. The experimental number for HDME at each concentration was 6, and for Ro 20-1724 was 4.

HDME (3~300 μM), similar to Ro 20-1724 (1~10000 nM), concentration-dependently displaced 2 nM [^3^H]-rolipram binding on HARBSs of guinea pig brain cell membranes (Figure 4A, B). The respective EC_50 _(PDE4_H_) values of HDME and Ro 20-1724 for displacing [^3^H]-rolipram binding were 106.6 ± 39.5 (*n *= 6) μM and 87.0 ± 29.0 (*n *= 4) nM. However, the IC_50 _values for inhibiting PDE4 catalytic activity of HDME and Ro 20-1724 were taken to be PDE4_L _values, which respectively were 3.0 and 8.7 μM. Thus, the PDE4_H_/PDE4_L _ratios of HDME and Ro 20-1724 were 35.5 and 0.01, respectively.

### Supression of airway hyperresponsiveness in vivo

**Figure 5:**
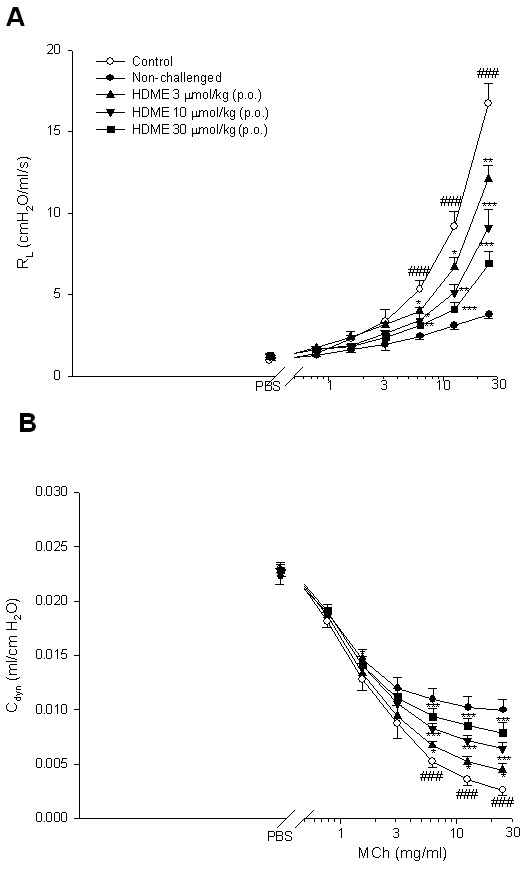
**Effects of HDME (3~30 µmol/kg, p.o.) on airway resistance**. Effects of HDME (3~30 µmol/kg, p.o.) on airway resistance (R_L_, A) and lung dynamic compliance (C_dyn_, B) in sensitized and challenged mice which received aerosolized methacholine (MCh, 0.78~25 mg/ml) 2 days after primary allergen challenge. ^### ^*P <*0.001, compared to the non-challenged group. * *p *< 0.05, ** *p <*0.01 and *** *p <*0.001, compared to the control (vehicle) group. The number of mice in each group was 10. PBS, phosphate-buffered saline.

R_L _values at the baseline for the control (vehicle), non-challenged, and 3, 10, and 30 μmol/kg HDME groups were 1.03 ± 0.03, 1.04 ± 0.05, 1.06 ± 0.05, 1.08 ± 0.06, and 1.09 ± 0.07 cmH_2_O/ml/s, respectively, and these values did not significantly differ from each other. R_L _values of PBS nebulization for each group were 1.05 ± 0.04, 1.06 ± 0.05, 1.10 ± 0.07, 1.06 ± 0.04, and 1.07 ± 0.06 cmH_2_O/ml/s, respectively, which also did not significantly differ from each other. Administration of nebulized PBS did not affect the R_L _values of the baseline in each group. However, MCh (6.25~25 mg/ml) concentration-dependently and significantly increased R_L _values (Figure 5A), and decreased C_dyn _values (Figure 5B) in the control sensitized and challenged group compared to the non-challenged group. HDME (3~30 μmol/kg, p.o.) significantly suppressed these changes (Figure 5).

**Figure 6:**
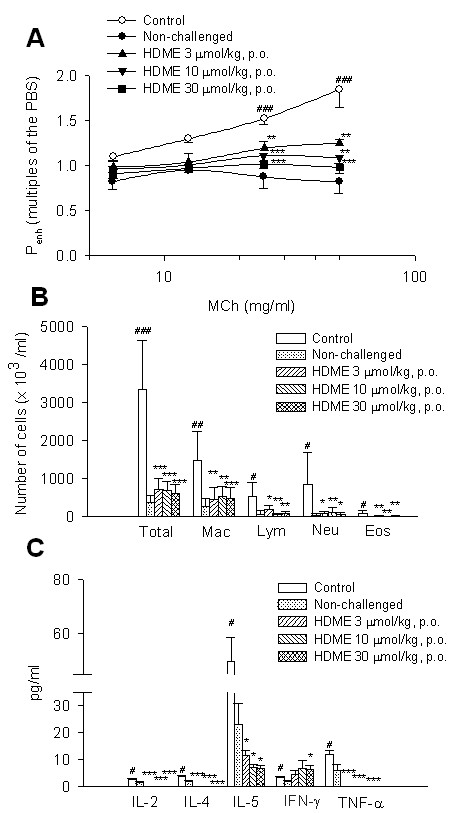
**Effects of HDME (3~30 μmol/kg, p.o.) on the enhanced pause (P_enh_)**. Effects of HDME (3~30 μmol/kg, p.o.) on the enhanced pause (P_enh_) (A), inflammatory cells (B), and cytokines (C) in sensitized mice which received aerosolized methacholine (6.25~50 mg/ml) 2 days after primary allergen challenge. ^# ^*p <*0.05, ^## ^*p <*0.01, and ^### ^*p <*0.001, compared to the non-challenged group. * *p <*0.05, ** *p <*0.01, and *** *p <*0.001, compared to the control (vehicle) group. The number of mice in each group was 10. Total, total cells; Mac, macrophages; Lym, lymphocytes; Neu, neutrophils; Eos, eosinophils; IL, interleukin; IFN, interferon; TNF, tumor necrosis factor.

P_enh _values at the baseline for the control (vehicle), non-challenged, and 3, 10, and 30 μmol/kg HDME groups were 2.32 ± 0.04, 2.41 ± 0.05, 2.45 ± 0.04, 2.36 ± 0.05, and 2.43 ± 0.03, respectively, and these values did not significantly differ from each other. P_enh _values with PBS nebulization for each group were 2.42 ± 0.05, 2.41 ± 0.04, 2.43 ± 0.05, 2.38 ± 0.06, and 2.44 ± 0.06, respectively, which also did not significantly differ from each other. Administration of nebulized PBS did not affect the P_enh _value of the baseline in each group. However, MCh (6.25~50 mg/ml) concentration-dependently increased P_enh _values from 1-fold with PBS exposure to 1.85 ± 0.20-fold in control sensitized and challenged mice (Figure 5A). P_enh _values of MCh at 25 and 50 mg/ml in control sensitized and challenged mice were significantly enhanced compared to those in non-challenged mice. HDME (3~30 μmol/kg, p.o.) dose-dependently and significantly attenuated the enhancement of P_enh _values induced by 25 and 50 mg/ml MCh (Figure 6A).

### Suppression of inflammatory cells and cytokines in the BALF

In this special animal model, the number of neutrophils in the bronchoalveolar lavage fluid of control sensitized and challenged mice was significantly more than that of eosinophils. The numbers of total inflammatory cells, macrophages, lymphocytes, neutrophils, and eosinophils from the BALF of control sensitized and challenged mice significantly increased compared to those of non-challenged mice (Figure 6B). HDME (3~30 μmol/kg, p.o.) significantly suppressed the increases in numbers of total inflammatory cells, macrophages, lymphocytes, neutrophils, and eosinophils (Figure 6B). Noticeably, the numbers of eosinophils were abolished by HDME at various doses used.

Compared to those in non-challenged mice, levels of cytokines, such as IL-2, IL-4, IL-5, IFN-γ, and TNF-α, in the BALF of control sensitized and challenged mice significantly increased (Figure 6C). HDME (3~30 μmol/kg, p.o.) also significantly suppressed increases in levels of IL-2, IL-4, IL-5, and TNF-α, but enhanced the level of IFN-γ at 30 μmol/kg (Figure 6C).

### Suppression of IgE and IgG_2a _in the serum and BALF

**Figure 7:**
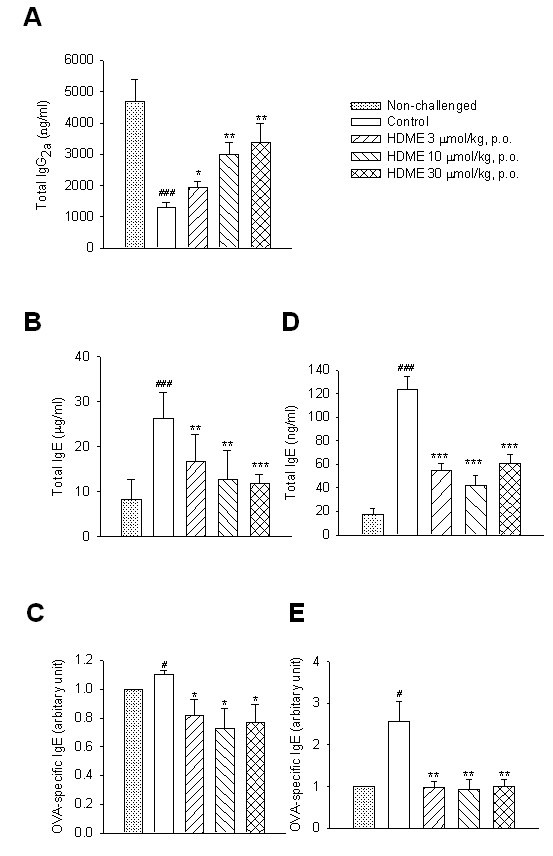
**Effects of HDME (3~30 μmol/kg, p.o.) on total IgG_2a _and IgE**. Effects of HDME (3~30 μmol/kg, p.o.) on total IgG_2a _(A), total IgE (B) and ovalbumin-specific IgE (C) levels in the serum and bronchial alveolar lavage fluid (D, E) of sensitized mice which had received aerosolized methacholine (6.25~50 mg/ml) 2 days after primary allergen challenge. ^# ^*p <*0.05, ^## ^*p <*0.01 and ^### ^*p <*0.001, compared to the non-challenged group. * *p <*0.05, ** *p <*0.01 and *** *p <*0.001, compared to the control (vehicle) group. Each value represents the mean ± SEM. The number of mice in each group was 10.

The level of total IgG_2a _in the serum of control sensitized and challenged mice was significantly supressed compared to that of non-challenged mice. HDME (3~30 μmol/kg, p.o.) dose-dependently and significantly enhanced this supression (Figure 7A). However, levels of total and OVA-specific IgE in the BALF and serum of control sensitized and challenged mice were significantly enhanced compared to those of non-challenged mice. HDME (3~30 μmol/kg, p.o.) dose-dependently and significantly suppressed these enhancements (Figure 7B-E).

### No effect on xylazine/ketamine-induced anesthesia

**Figure 8:**
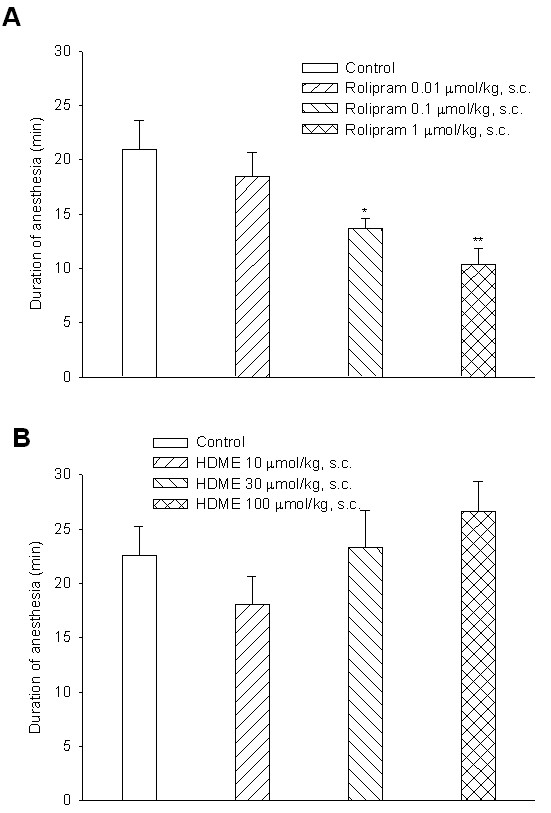
**Effects of subcutaneously administered rolipram (A) and HDME (B)**. Effects of subcutaneously administered rolipram (A) and HDME (B) on the duration of xylazine (10 mg/kg, i.p.)/ketamine (70 mg/kg, i.p.)-induced anesthesia in mice. Rolipram was administered 0.25 h and HDME 1 h before anesthesia. ** *p <*0.01, *** *p <*0.001, compared to the vehicle (control). Each value represents the mean ± SEM. The number of mice in each group was 10.

The durations of xylazine/ketamine-induced anesthesia in control (vehicle) mice for the rolipram- and HDME-treated groups were 22.0 ± 3.0 (*n *= 10) and 22.4 ± 1.5 min (*n *= 10), respectively. Rolipram (0.01~1 μmol/kg, s.c.) dose-dependently shortened the duration, and at doses of 0.1 and 1 μmol/kg (s.c.) significantly shortened the duration (Figure 8A). In contrast, HDME (10~100 μmol/kg, s.c.) did not significantly influnce the duration (Figure 8B).

## Discussion

Allergic asthma (type-I allergic airway disease) is a chronic respiratory disease characterized by AHR, mucus hypersecretion, bronchial inflammation, and elevated IgE levels. Th2 cells, together with other inflammatory cells such as eosinophils, B cells, and mast cells are thought to play critical roles in the initiation, development, and chronicity of this disease [27]. This clinical definition fails to account for the atypical and often more severe phenotype found in a considerable proportion of asthmatics who have increased neutrophil cell counts in the airway as a distinguishing trait. Neutrophilic inflammation is a hallmark of another type of allergic airway pathology, hypersensitivity pneumonitis. Considered as an immune counterpart of asthma, hypersensitivity pneumonitis is a prototypical type-III allergic inflammatory reaction involving the alveoli and lung interstitium, steered by Th1 cells and IgG and, in its chronic form, accompanied by fibrosis [28]. Thus, this animal model appears to be suitable for studying the effects of drugs on the atypical asthma and COPD, and for screening those on typical asthma. One hypothesis emphasizes an imbalance in Th cell populations favoring expression of Th2 over Th1 cells in typical asthma. Cytokines released from Th2 cells are IL-4, IL-5, IL-6, IL-9, and IL-13, and those from Th1 cells are IL-2, IL-12, IFN-γ, and TNF-α [29,30]. In the present results, HDME (3~30 μmol/kg, p.o.) significantly decreased R_L _(Figure 5A), and increased C_dyn _(Figure 5B), and also attenuated P_enh _values (Figure 6A) suggesting that it significantly suppresses AHR. The numbers of all types of inflammatory cells examined, including total inflammatory cells, macrophages, lymphocytes, neutrophils, and eosinophils in the BALF of sensitized and challenged mice were reduced by HDME (3~30 μmol/kg, p.o.) (Figure 6B). It is well known that after oral administration and digestion of hesperidin, a flavanone glycoside comprised of the flavanone hesperetin and the disaccharide rutinose, forms hesperetin. Similarly, hesperetin is also formed by demethylation of HDME after oral administration. However, whether the effects of HDME on lung tissue are similar to those of hesperidin [9] needs to be further investigated. It also suppressed levels of IL-2, IL-4, IL-5, and TNF-α, but significantly enhanced the level of IFN-γ (Figure 6C). These results suggest that HDME fully suppresses Th2 cells and partially activates Th1 cells, and ameliorates this imbalance occurred in typical asthma. However, this partial activation of Th1 cells may offset, at least in a part, some anti-inflammatory effects of HDME, by which IL-2 and TNF-α released from Th1 cells were reduced. However, the number of neutrophils was significantly reduced by HDME, suggesting that it may have a benefit for treating atypical asthma. Similarly, the numbers of macrophages and neutrophils were reduced by HDME, suggesting that it may ameliorate COPD too.

IL-4 and IL-13 were shown to induce AHR in mouse asthma models [31,32]. IL-4 has three primary effects. First, IL-4 promotes B cell differentiation to plasma cells that secrete antigen-specific IgE antibodies. Second, IL-4 promotes mast cell proliferation. Third, increased IL-4 upregulates endothelial cell expression of adhesion molecules for eosinophils [33]. IL-5 mobilizes and activates eosinophils, leading to the release of a major basic protein, cysteinyl-leukotriene, and eosinophil peroxidase that contribute to tissue damage and AHR [32,34]. Phosphoinositide 3-kinase δ (p110δ) was shown to play a crucial role in the development, differentiation, and antigen receptor-induced proliferation of mature B cells [35,36], and inhibition of p110δ attenuates allergic airway inflammation and AHR in a murine asthma model [35,37]. In addition, IL-4 and IL-13 are important in directing B cell growth, differentiation, and secretion of IgE [38]. However, IFN-γ released from Th1 cells preferentially directs B cell switching of IgM to IgG_2a _and IgG_3 _in mice [39,40]. HDME (3~30 μmol/kg, p.o.) herein dose-dependently and significantly enhanced total IgG_2a _level in the serum and suppressed total and OVA-specific IgE levels in the BALF and serum of sensitized and challenged mice, suggesting that HDME has immunoregulatory and antiallergic asthmatic effects.

In the present results, HDME selectively inhibited PDE4 activity with the IC_50 _and K_i _values of 3.0 and 2.1 μM, respectively. Selective PDE4 inhibitors specifically prevent the hydrolysis of cAMP, a 3',5'-cyclic nucleotide, and therefore have broad anti-inflammatory effects such as inhibition of cell trafficking and of cytokine and chemokine release from inflammatory cells. The increased cAMP levels induced by these selective PDE4 inhibitors subsequently activate cAMP-dependent protein kinase which may phosphorylate and inhibit myosin light-chain kinase, thus inhibiting contractions [41]. The precise mechanism through which relaxation is produced by this second-messenger pathway is not known, but it may result from decreased intracellular Ca^2+ ^([Ca^2+^]_i_). The decrease in [Ca^2+^]_i _may be due to reduced influx of Ca^2+^, enhanced Ca^2+ ^uptake into the sarcoplasmic reticula, or enhanced Ca^2+ ^extrusion through cell membranes [41]. Thus selective PDE4 inhibitors may have bronchodilatory effects. The second-generation PDE4 inhibitors, cilomilast and roflumilast, have reached the clinical trial stage and exhibit some beneficial effects in treating asthma and COPD [42]. The effectiveness of these PDE4 inhibitors may be limited by their clinical potency when using doses that have minimal adverse effects such as headaches, diarrhea, nausea, and abdominal pain. The PDE4_H_/PDE4_L _ratios of cilomilast and roflumilast were respectively reported to be 117.8 nM/120 nM (1), and 2.4 nM/0.8 nM (3) [21,43], which are considerably greater than that (0.01~0.001) of rolipram [7]. Owing to its adverse effects or lack of efficacy, cilomilast was discontinued for use against asthma after phase II clinical trials in 2003 [42]. In terms of tolerability over 6 months with 15 mg twice daily for COPD in a phase III study, cilomilast was reported to be associated with higher frequencies of diarrhea and nausea than a placebo [42]. Roflumilast was evaluated for asthma and COPD in phase III clinical trials, and was reported to reduce those adverse effects after longer-term treatment at 0.5 mg once daily [42]. Roflumilast, compared to a placebo, was reported to significantly improve the mean pre- and post-bronchodilator forced expiratory volumes in 1 s (FEV_1_) in patients with moderate-to-severe COPD. However, nausea, diarrhea, weight loss, and headaches were more frequent in patients in the roflumilast group. These adverse events were associated with increased patient withdrawal [44,45]. Recently, roflumilast was approved by the European Commission as an add-on to bronchodilator therapy for maintenance treatment of severe COPD associated with chronic bronchitis in adults with a history of frequent exacerbations. However, the US Food and Drug Administration voted against using roflumilast to treat COPD. The PDE4_H_/PDE4_L _ratio of AWD 12-281, another selective PDE4 inhibitor, was reported to be 104 nM/9.7 nM (approximately 11) [46]. AWD 12-281 was undergoing clinical development phase IIa trials for COPD, and was reported to be a unique potential drug for the topical treatment of asthma and COPD [47]. AWD 12-281 was reported to be a very promising drug candidate for treating lung inflammation when administered by inhalation and for treating atopic dermatitis [48]. However, AWD-12-281 was also discontinued in clinical trials for both asthma and COPD owing to a lack of efficacy [49,50]. Many compounds that are in development will not reach the market as monotherapies unless their emetic liability is reduced [51], although inhaled GSK256066 demonstrated efficacy in trials in asthma [52] and oral apremilast was clinically reported to be effective for treating severe plaque-type psoriasis [53]. PDE4 subtypes (A~D) may be considered for drug development of new PDE4 inhibitors. PDE4D inhibition in non-target tissues promotes emesis, since PDE4D knock-out mice showed reduction of xylazine/ketamine-triggered anesthesia which is used as a surrogate marker for emesis in mice, a non-vomiting species [13]. Recently, small-molecule allosteric modulators of PDE4D that do not completely inhibit enzymatic activity were reported to reduce emesis and have therapeutic benefits of a brain distribution, for such entities as Alzheimer's disease, Huntington's disease, schizophrenia, and depression [54]. In contrast to PDE4D, selective inhibition of PDE4A and/or PDE4B in proinflammatory and immune cells is believed to evoke the therapeutically desired effects of these drugs [55]. Cilomilast has a higher potency for PDE4D compared to PDE4A and PDE4B, while roflumilast is non-selective for these four PDE4 subtypes with similar degrees of inhibition [56]. There is no literature about AWD 12-281's inhibition of PDE4 subtypes until now. However, whether HDME selectively inhibits the PDE4 subtype also needs to be further investigated.

In the present results, the PDE4_H_/PDE4_L _ratio of HDME was calculated to be 35.5, which is considerably greater than that of AWD 12-281. In addition, HDME did not influnce xylazine/ketamine-induced anesthesia. However, rolipram, a selective PDE4 inhibitor, reversed the anesthesia. The reversing effect may occur through presynaptic α_2_-adrenoceptor inhibition [57], because MK-912, an α_2_-adrenoceptor antagonist, was reported to reverse xylazine/ketamine-induced anesthesia in rats [12] and trigger vomiting in ferrets [57]. In contrast, clonidine, an α_2_-adrenoceptor agonist, prevented emesis induced by PDE4 inhibitors in ferrets [57]. The present results also suggest that HDME may have few or no adverse effects, such as nausea, vomiting, and gastric hypersecretion.

## Conclusions

**Figure 9:**
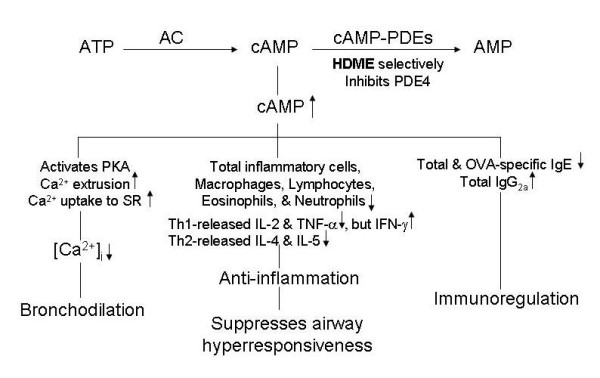
**Mechanisms of action of HDME**. HDME selectively inhibits PDE4 activities and results in an increase in cAMP, which activates cAMP-dependent protein kinase (PKA) and increases calcium extrusion from the intracellular space and uptake to sarcoplasmic reticula (SR). Therefore, HDME largely decreases the concentration of intracellular calcium ([Ca^2+^]_i_) and results in bronchodilatation. The increase in cAMP also has anti-inflammatory and immunoregulatory effects. AC, adenylate cyclase; Th, T-helper cells; Ig, immunoglobulin; IL, interleukin; IFN, interferon; TNF, tumor necrosis factor. Up and down arrows respectively indicate increases and decreases.

In conclusion, HDME exerted anti-inflammatory effects, including suppression of AHR, and reduced expressions of inflammatory cells and cytokines in this murine model, which appears to be suitable for studying the effects of drugs on atypical asthma and COPD, and for screening those on typical asthma. Its mechanisms are summarized in Figure 9. However, HDME did not influnce xylazine/ketamine-induced anesthesia. Thus, HDME may have the potential for use in treating typical and atypical, at least in part, asthma, and COPD.

## Abbreviations

AHR: airway hyperresponsiveness; cAMP: adenosine 3',5' cyclic monophosphate; cGMP: guanosine 3',5' cyclic monophosphate; COPD: chronic obstructive pulmonary disease; DMSO: dimethyl sulfoxide; EDTA: ethylenediaminetetraacetic acid; HARBSs: high-affinity rolipram-binding sites; HDME: hesperetin-7,3'-*O*-dimethylether; IFN: interferon; Ig: immunoglobulin; IL: interleukin; K_i_: dissociation constant for inhibitor binding; MCh: methacholine; PBS: phosphate-buffered saline; PDE: phosphodiesterase; PDE4_H_: high affinity for PDE4; PDE4_L_: low affinity for PDE4; P_enh_: enhanced pause; PMSF: phenylmethanesulfonyl fluoride; Ro 20-1724: 4-(3-butoxy-4-methoxybenzyl)-2-imidazolidinone; TCM: traditional Chinese medicine; Th: T-helper; TNF: tumor necrosis factor.

## Competing interests

The authors declare that they have no competing interests.

## Authors' contributions

YLY conceived of the study and participated in its design and coordination. HTH and KHW were responsible for carrying out the data analysis, and the construction of figures. CYH was responsible for carrying out the majority of in vivo and in vitro studies, CnMC was responsible for counting the numbers of inflammatory cells, ChiMC was responsible for synthesizing HDME. WCK conceived of and contributed to the design of the studies, supervised data analysis and contributed to writing and editing the final manuscript. All authors read and approved the final manuscript.
